# Surface-Enhanced Raman Spectroscopy (SERS) for Identifying Traces of Adenine in Organic-Bearing Extraterrestrial Dust Analogs Captured in the Tanpopo Aerogel after Hypervelocity Impacts

**DOI:** 10.3390/gels10040249

**Published:** 2024-04-06

**Authors:** Aline Percot, Farah Mahieddine, Hajime Yano, Sunao Hasegawa, Makoto Tabata, Akihiko Yamagishi, Hajime Mita, Alejandro Paredes-Arriaga, Marie-Christine Maurel, Jean-François Lambert, Donia Baklouti, Emilie-Laure Zins

**Affiliations:** 1 Sorbonne Université, CNRS MONARIS UMR8233, 75005 Paris, France; farahmahieddine5@gmail.com (F.M.); emilie-laure.zins@sorbonne-universite.fr (E.-L.Z.); 2 Institute of Space and Astronautical Science, Japan Aerospace Exploration Agency, Sagamihara 252-5210, Japan; yano.hajime@jaxa.jp (H.Y.);; 3 Department of Physics, Chiba University, Chiba 263-8522, Japan; makoto@hepburn.s.chiba-u.ac.jp; 4 Department of Applied Life Science, Tokyo University of Pharmacy and Life Sciences, Hachiojishi 192-0392, Japan; yamagish@toyaku.ac.jp; 5 Fukuoka Institute of Technology, Wajiro-higashi, Fukuoka 811-0295, Japan; mita@fit.ac.jp; 6 Posgrado en Ciencias de la Tierra, Universidad Nacional Autónoma de México, Circuito Exterior S/N, Cd. Universitaria, Coyoacán, C.P, Ciudad de México 04510, Mexico; alejandro.paredes@correo.nucleares.unam.mx; 7 Laboratorio de Evolución Química, Departamento de Química de Radiaciones y Radioquímica, Instituto de Ciencias Nucleares, Universidad Nacional Autónoma de México, Circuito Exterior S/N, Cd. Universitaria, Coyoacán, C.P, Ciudad de México 04510, Mexico; 8 Institut de Systématique, Evolution, Biodiversité( ISYEB), Museum National d’Histoire Naturelle (MNHN), CNRS, Sorbonne Université, EPHE, Université des Antilles, 57 rue Cuvier, CP 51, 75005 Paris, France; marie-christine.maurel@sorbonne-universite.fr; 9 Laboratoire d’Archéologie Moléculaire et Structurale, Sorbonne Université, CNRS UMR 8220, 75005 Paris, France; jean-francois.lambert@sorbonne-universite.fr; 10 Institut d’Astrophysique Spatiale, Université Paris-Saclay, CNRS, 91405 Orsay Cedex, France

**Keywords:** surface-enhanced Raman spectroscopy (SERS), adenine, Creighton’s colloid, aggregating salt, trace analysis, aerogel, hypervelocity impacts, serpentinite dust

## Abstract

Raman spectroscopy is a non-destructive analytical technique for characterizing organic and inorganic materials with spatial resolution in the micrometer range. This makes it a method of choice for space-mission sample characterization, whether on return or in situ. To enhance its sensitivity, we use signal amplification via interaction with plasmonic silver-based colloids, which corresponds to surface-enhanced Raman scattering (SERS). In this study, we focus on the analysis of biomolecules of prebiotic interest on extraterrestrial dust trapped in silica aerogel, jointly with the Japanese Tanpopo mission. The aim is twofold: to prepare samples as close as possible to the real ones, and to optimize analysis by SERS for this specific context. Serpentinite was chosen as the inorganic matrix and adenine as the target biomolecule. The dust was projected at high velocity into the trapping aerogel and then mechanically extracted. A quantitative study shows effective detection even for adenine doping from a 5·10^−9^mol/L solution. After the dust has been expelled from the aerogel using a solvent, SERS mapping enables unambiguous adenine detection over the entire dust surface. This study shows the potential of SERS as a key technique not only for return samples, but also for upcoming new explorations.

## 1. Introduction

It is hypothesized that an important quantity of organic matter could have been delivered to the early Earth via comet and asteroid bombardment [1,2]. Studying primitive extraterrestrial dust related to these small bodies is thus crucial to better characterize and understand this exogenous delivery that could have played a key role in prebiotic chemistry.

The Tanpopo 1 and 2 experiments were the first Japanese astrobiology missions conducted on board the International Space Station at 500 km altitude of the low Earth orbit in 2015–2020 [3]. The Tanpopo 1 and 2 missions consisted of several subthemes: the exposure and possible capture of terrestrial microbes in space, intact capture and post-retrieval analysis of organic compounds in interplanetary dust, exposure of astronomical organic analog compounds in space, measurement of micrometeoroid and space debris flux in the ISS orbit, and evaluation of ultralow-density aerogel developed for the micrometeoroid capture experiment. For the capture experiments, Tanpopo capture panels were employed. They consist of blocks of amorphous silica aerogel of 0.01 g/cc bulk density that were exposed to collect hypervelocity impact microparticles. Possible captured particles may include extraterrestrial dust (similar to micrometeorites and Interplanetary Dust Particles (IDPs) collected after entry in the Earth atmosphere), artificial orbital debris, and possibly terrestrial microparticles if they can reach the low Earth orbit altitudes. After collecting and returning the captured dust particles to the ground laboratory at the Institute of Space and Astronautical Science, Japan Aerospace Exploration Agency (ISAS/JAXA), the captured particles were searched, documented, characterized, and extracted from the aerogel panels and then allocated for detailed analyses including the organic chemistry, isotope compositions, mineralogy, and impact-track morphology of the samples by the state-of-art analytical technique.

In this study, we developed an alternative sample analysis procedure to chemically analyze the organic matter in hypervelocity impact meteoroid analogs embedded within the Tanpopo aerogel. It was therefore important to optimize the analysis process on realistic samples that also reproduce the collection process in Tanpopo aerogel. Thus, as a ground-based simulation for the Tanpopo missions, we have conducted hypervelocity impacts experiments for the aerogel capture of rock powders at 6 km/s using a two-stage light gas gun (LGG) at ISAS/JAXA, in order to evaluate the extent of modification of organic matter in micrometeoroids and to develop a non-destructive method of organic matter analysis in this context. 

The so-called “primitive” extraterrestrial dust originates from small bodies, such as asteroids and comets, which did not undergo differentiation processes after their accretion. This type of dust particle constitutes the main type of extraterrestrial dust collected on Earth. Decades of studies in this field, based mainly on meteorites of the chondritic type and on IDPs and micrometeorites of the same category, show that they are complex materials, characterized by relatively high mineralogical and compositional heterogeneities at the micrometric scale. This heterogeneity is mainly a heritage of the dust and ice particles that formed in the protoplanetary disk and got mixed and accreted to form planetesimals [4,5]. Post-accretion processes on the parent body, such as hydrothermal alteration and metamorphism, are responsible for a certain diversity of composition between small bodies and different stages of mineralogical and chemical evolution that can be observed from the meter (=asteroid scale) to the sub-millimeter scale (dust particle scale) [6]. As a result, in chondritic dust, different phases are observed close to each other or intermingled at the sub-micrometer scale (e.g., amorphous and crystalline phases, minerals, and carbonaceous materials [7,8]), with sometimes different formations and evolution histories (e.g., highly crystalline minerals possibly formed in the inner and hot part of the protosolar disk, mixed with organic compounds and volatiles, form the external and cold region of the disk) [9,10]. In chondrites (=primitive meteorites) that underwent aqueous alteration, phyllosilicates are dominant matrix components. Among them, the majority are close in composition and structure to terrestrial serpentines (iron and magnesium-rich T-O type silicates), although some T-O-T silicates such as saponites can also be found. For this reason, we chose a lizardite serpentine as a model mineral phase for our experiments. This has the additional advantage of allowing a comparison with systems extant on the early Earth (and early Mars), where such phases were formed from ultramafic rocks containing olivine.

In this study, we describe the results of LGG experiments at ~6 km/s, which is near the upper limit of the LGG velocity range but still at the lower end of the impact velocity distribution of micrometeoroids in the Earth vicinity. We used lizardite particles (50–100 μm) doped with organic molecules to document resistance to LGG acceleration and test the extraction and detection conditions for organic matter. Although the impacting velocity of particles on ISS may be much higher, we tested the highest practical velocity with the LGG employed to obtain the baseline data to test the possible capturing ability of the aerogel. As a first step, adenine, a nucleobase, was chosen to dope the mineral dust. Indeed, adenine is a key molecule in exobiology/astrobiology research because it is a component of nucleotides and a significant prebiotic factor [11]. It has also been detected in different chondrite samples (mainly, but not only, Orgueil and Murchison) in sub-ppm amounts [12,13,14]. 

Moreover, among nucleobases, adenine is particularly photo-stable. Adenine is involved in many biological processes, and in particular, as a metabolic cofactor in adenosine triphosphate (ATP), adenosine diphosphate (ADP), adenosine monophosphate (AMP), nicotinamide adenine dinucleotide (NAD), nicotinamide adenine dinucleotide phosphate (NADP), flavine adenine dinucleotide (FAD), coenzyme A (CoA), cyclic adenosine monophosphate (cAMP), as well as in DNA and RNA. Adenine was chosen as the first-intention test molecule.

Some of the main interests in the analysis of meteorites and cosmic dust are the geochemical characterization and the detection and characterization of organic molecules. Several techniques can be used to characterize the captured samples in the aerogel, but many of them, such as gas chromatography, require extensive sample preparation and treatment. Raman spectroscopy is one of the most popular techniques for analyzing non-terrestrial samples because the samples do not require any pretreatment and provide structural and molecular information [15]. Micro-Raman spectroscopy allows in situ analyses, limiting contamination. Surface-enhanced Raman spectroscopy (SERS) is a highly sensitive method for detecting traces of organic molecules [16,17,18,19]. SERS was previously used to study how adenine adsorbed on specimens from the Murchison and Zagami meteorites and monitor the driving forces that regulate the binding of nucleic bases to such extraterrestrial rocks [20]. This technique provides signal enhancements between 10^5^ and 10^9^—fold [21]. For example, this technique has been used to improve the Limit of Detection (LOD) of maleimide, an N-containing heterocyclic molecule common in biochemical processes, with potential applications in astrobiology [22]. SERS has been used to identify adenine in experiments with a Martian meteorite [23], calcite, clay, and basalt [24], and even to search for molecular evidence of life in rocks, sediments, and sedimentary deposits [21]. 

In this article, we prepared serpentinite dust samples and enriched them with adenine. These dusts were then projected at a velocity of 6 km/s using a two-stage LGG into the aerogel used for trapping on the ISS. Raman and SERS were used to characterize these samples before projection and after projection/extraction steps. Silver Creighton colloids were used to amplify the Raman signal [25]. This method has already been successfully used to detect the adenine adsorbed on Zagami, a Martian meteorite, and on Murchison, on montmorillonite samples, and also trapped directly in silica aerogel [20,26,27]. 

## 2. Results

To assess the reliable use of SERS for return samples analysis or for the in-situ search of organic matter in space missions, preliminary analyses on model samples as close as possible to the real specimens are essential. An important part of this work therefore concerns the development of model samples, involving the choice of an inorganic matrix, organic molecules to be detected, and high-velocity trapping in the capture aerogel. All these choices and sample preparation conditions are detailed in the Materials and Methods section. The second part of this work concerns the optimization of the analysis of these model samples by SERS.

To optimize the analysis protocol, the analysis was performed on clusters in a first step and on individual dust in a second step. To perform SERS analysis on 50 μm diameter particles is very challenging, because of the small size of the particle and the heating risk.

### 2.1. Analysis of Adenine-Doped Clusters

**Figure 1:**
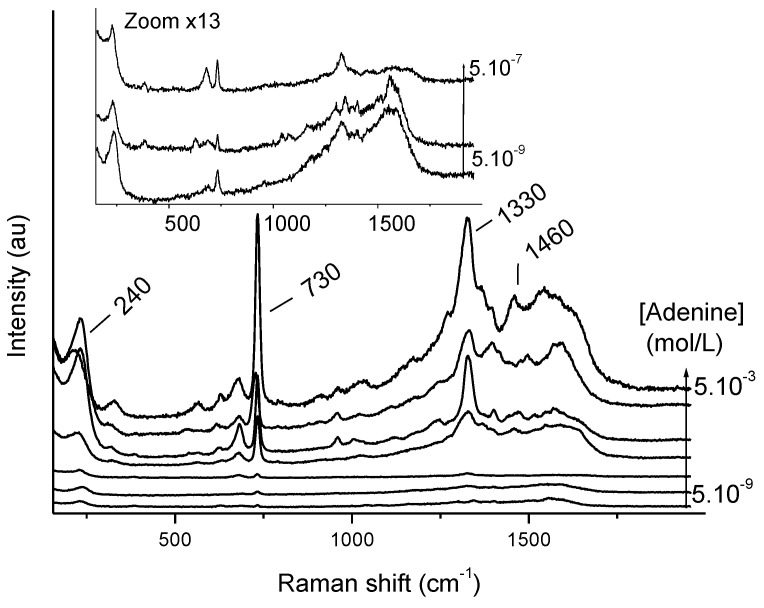
SERS spectra as a function of adenine concentration after incubation with serpentine dust. Measurements are taken on a cluster of grains. Representative spectra are shown.

The SERS spectra of serpentinite dust particle clusters doped with more or less concentrated aqueous solutions according to the protocol presented in the Materials and Methods section are presented in Figure 1. It is worth noting that the way the samples are prepared can lead to the mesoscopic-level heterogeneity of the particles, with adenine possibly accumulating in certain places. This is due to adenine desorption upon contact with the silver colloid suspension, followed by uneven drying (“coffee-ring” effect). The quantities of adenine introduced are therefore not necessarily representative of the quantity actually probed by micro-Raman spectroscopy. In Table S1, the maximum adenine loading is estimated. Despite the complexity of this solid-phase measurement on heterogeneous clusters, the adenine signature can be found on all spectra presented in Figure 1, the intensity of the signal being correlated with the concentration of the initial solution. Even when doped with a very dilute solution (5·10^−9^ mol/L), adenine was clearly detected in the dusts. The band at 730 cm^−1^ was attributed to a breathing mode, and the multi-component band at 1330 cm^−1^ to mixed in-plane stretching vibrations of the six-membered ring [27].

The band at 230 cm^−1^ is assigned to silver chloride adsorption. To optimize adenine detection, MgCl_2_ was added by registering SERS spectra as a function of MgCl_2_ concentration (data not shown). The final chosen MgCl_2_ concentration was 10^−2^ mol/L. 

### 2.2. Analysis of Individual Doped Serpentinite Dust 

Individual dusts were analyzed by SERS prior to projection into the aerogel. To perform SERS analysis on individual dust, the particles are deposited on a golden mirror and a drop of aqueous premix is deposited on one individual particle. The main difficulty is to locate the particle after the colloid addition. Mapping is therefore carried out to localize the particle in the drop. 

After a few minutes, the SERS spectrum appears. The characteristic adenine signature was obtained for the two concentrations used for enrichment (Figure S1). The signal is stable for around 30 min (as previously observed with clusters). The SERS spectra are identical to those obtained for the clusters (Figure 1). This promising step allows us to move on to the study of sieved and doped particles projected into the aerogel using the LGG, in order to reproduce mission return samples as closely as possible.

### 2.3. Analysis of Individual Doped Dust Infused in Adenine Solutions and Projected into the Aerogel after Mechanical Extraction

**Figure 2:**
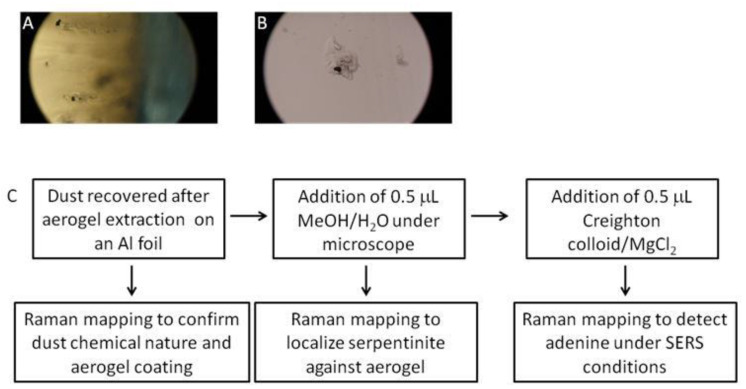
Captured particles in aerogel after projection at a velocity of 6.2 km/s using a two-stage LGG (**A**); particle after extraction with a small amount of aerogel around (**B**); sequence followed for the analysis of dust extracted from aerogel after LLG projection: observation, extraction of aerogel by addition of MeOH/H_2_O, control Raman mapping and finally, addition of Creighton colloid for SERS analysis and adenine detection (**C**).

After LGG projection, dust are buried in aerogel (Figure 2).

**Figure 3:**
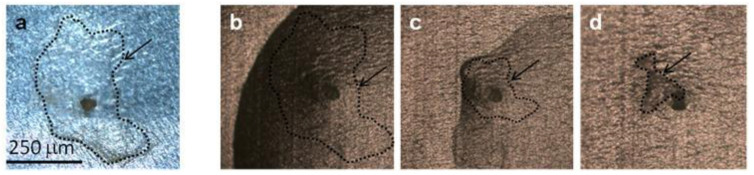
Monitoring of dust expulsion from the residual aerogel by addition of MeOH/H_2_O under microscope as a function of time (objective 10×): 50 μm serpentinite dust with remaining aerogel (arrow) (**a**), after addition of 0.5 μL MeOH/H_2_O (**b**), aerogel shrinking and solvent evaporation (**c**), dust expulsion with shrunken remaining aerogel (**d**).

Whatever the method of mechanical aerogel extraction, a thin layer of aerogel remains around the captured dust particle (Figure 3a). The presence of aerogel complicates the analysis of any organic molecules present, whatever the analytical technique. However, it seemed fundamental to us to be able to carry out an analysis of these dusts/particles without dissolving the aerogel, to limit any chemical alteration or modification of the dust content, and to be able to map any organic matter present as it would be essential to do for the dust collected by the Tanpopo mission. 

**Figure 4:**
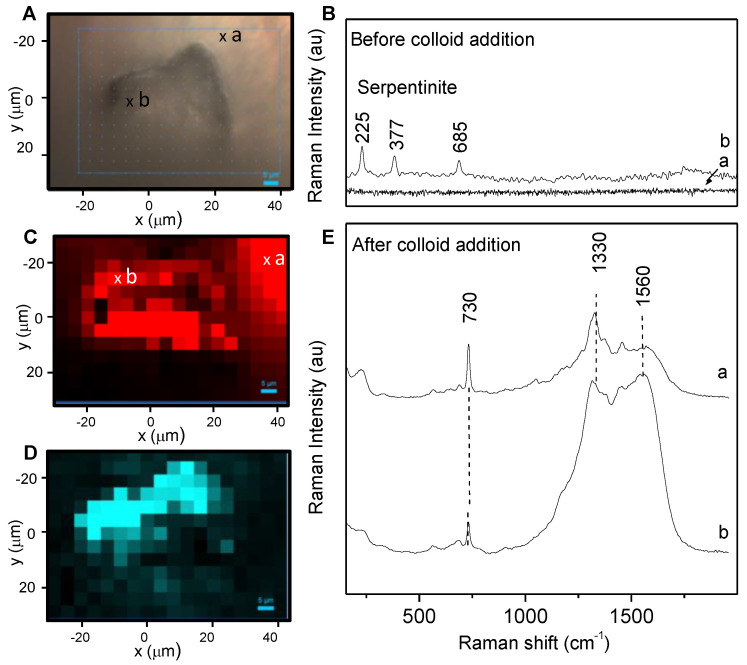
Raman mapping of a doped serpentinite dust with 5·10^−6^ mol/L of adenine after projection/extraction of the aerogel. (**A**) Light microscope image of the serpentinite dust after MeOH/H_2_O step, with the Raman map area; (**B**) representative Raman spectra extracted from Raman mapping out of the dust on the aluminum foil (a) and on the dust (b); (**C**,**D**) after Creighton colloid/MgCl_2_ addition, a new Raman mapping was performed, (**C**,**D**) maps correspond, respectively, to the distribution of the 730 cm^−1^ peak area (the red scale goes from 200 to 2000 counts) and of the ratio of the 1330 cm^−1^ peak area over the 730 cm^−1^ peak area (blue scale goes from 1 to 5). (**E**) Presents representative SERS spectra obtained out of the dust on the aluminum foil (a); on the dust (b). The complete set of spectra is presented in Figure S3.

As the first step, serpentinite and aerogel are mapped by Raman spectroscopy (Figure S2). Then, the presence and hydrophobicity of the Tanpopo aerogel have to be reduced to allow the addition of the aqueous colloid and then the contact with organic molecules that are essential for Raman signal exaltation and further detection. To this end, a drop of methanol/water solution is deposited on the aerogel. This causes the aerogel to shrink, literally “pushing” the dust out of the aerogel (Figure 3c,d). A first Raman map is then performed to check the absence of aerogel and the chemical nature of the dust. During LGG projection, debris and powder could be trapped in the aerogel and optically resemble serpentinite dust. Raman analysis confirms that the dust is composed in a majority of serpentinite (Figure 4B and Figure S2). No adenine signature was detected under Raman analysis. Then, a drop of colloidal solution can be added. After these steps, the particle is again mapped by micro-Raman spectroscopy (Figure 2C).

The Raman maps are shown in Figure 4, together with representative spectra. Prior to colloid addition, only a serpentinite signature can be detected (Figure 4B). After colloid addition, two different signatures are obtained for adenine: their location and a representative spectrum of each are presented in Figure 4C,E. These two adenine spectra are characterized by the band at 730 cm^−1^ and the two bands around 1330 and 1560 cm^−1^. They can be distinguished from each other by the relative intensity of these three bands, as well as by their location relative to the dust; the first signature (Figure 4E, spectrum b) is located on the dust, and could be attributed to the adenine adsorbed on the dust particle, whereas the second signature (Figure 4E, spectrum a) is located in the vicinity of the dust and could be due to the “free” adenine which redissolved in the aqueous phase during analysis (see Figure S3 for all the spectra obtained). The red color on the map presented in Figure 4C corresponds to the adenine distribution, whatever the signature is, as it monitors the area of the peak at 730 cm^−1^. The map presented in Figure 4D shows the ratio of the 1330 cm^−1^ peak area over the 730 cm^−1^ peak area. The intense blue color is localized on the particle (and not in its surroundings). This underlines the fact that the signature shown in Figure 4Eb (with the band at 1330 and 1560 cm^−1^ being more intense) is mainly located on the dust particle. This signature can be attributed to the chemical changes associated with a specifically adsorbed form of adenine interacting with the inorganic phase, and/or to more intense local heating, for this population of adenine molecules than for the ones that easily desorb, and consequently must have a weaker interaction with the surface. 

**Figure 5:**
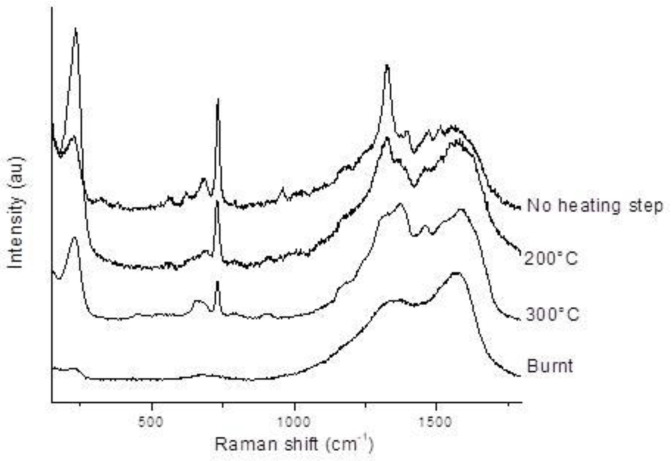
SERS spectra as a function of heating for a 10^−4^ mol/L adenine-doped serpentinite dust. The “burnt” adenine spectrum is obtained on doped serpentinite dust under laser excitation (with increased power). Measurements were taken on a cluster of grains. Representative spectra are shown.

Spectrum b seems to be a combination of adenine and carbon amorphous spectra (burnt adenine spectrum, Figure 5). The latter spectrum was obtained on the adenine burnt on serpentinite dust under laser excitation (with increased power) and shows characteristic broad bands at ~1350 and ~1560 cm^−1^, commonly associated to the D and G bands for disordered carbonaceous materials [28,29]. These observations can be related to the SERS analysis performed on heated samples (Figure 5), which shows the same pattern evolution. Indeed, after ~300 °C, adenine is degraded (Figure S4).

We analyzed four doped dusts projected into the aerogel using the LGG for this study, and relatively similar results were obtained for each, with two families of adenine spectra, as already observed. It should be noted, however, that no direct link can be made between the concentration of the solution used to dope the serpentinite with adenine and the SERS response of the individual dust particles, as adenine doping can be spatially heterogeneous on individual particles, as mentioned above, at least in the state in which they are analyzed. In one case, a signature of burnt organic matter was obtained, which may be due to the surface heating of the particle during its projection into the aerogel. The same phenomenon is likely to occur in real samples, i.e., micrometeoroids captured by aerogel in space, with surface carbonization.

**Figure 6:**
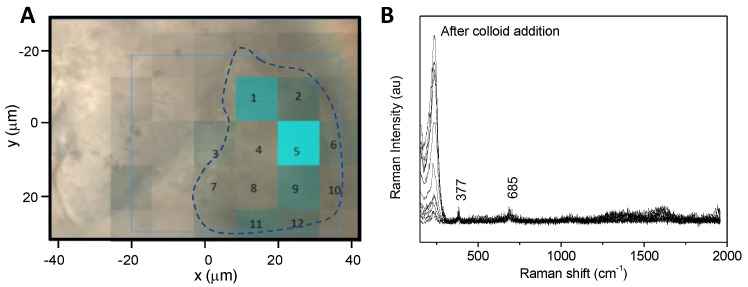
Raman mapping of a non-doped serpentinite dust after projection/extraction of the aerogel. (**A**) Light microscope image of the serpentinite dust (blue line to highlight the contour) after MeOH/H_2_O step and Creighton colloid/MgCl_2_ addition, superimposed with a Raman map; blue map corresponds to the area of the serpentinite 685 cm^−1^ band; (**B**) all the spectra obtained on the dust surface after colloid addition are presented. No signal corresponding to adenine markers, at 730 and 1330 cm^−1^, were detected all over the surface mapped.

Un-doped serpentinite samples were also prepared (with projection using the LGG step) and analyzed as a control (Figure 6). No adenine could be detected in these samples, even with optimized SERS conditions. Only the spectrum of serpentinite could be observed.

This analytical sequence involving SERS spectroscopy as an original detection method enables the search for organic matter in the particles collected by aerogel, with a maximum protection of the organic matter on the surface of the particles trapped in the aerogel. One of the advantages of SERS is that it does not require chemical extraction (with or without acid hydrolysis). Moreover, this analysis method is applicable to very small particle sizes, between 50 and 100 μm in this study, with chemical analysis of the particle matrix. The high sensitivity of the detection, up to single molecule detection, is perfectly relevant to this context.

## 3. Conclusions

We have proposed herein a protocol for preparing analogs of the micrometeoroids trapped in aerogels used for exposition on space stations. Analysis of these dust particles without the chemical attack of the hydrophobic, amorphous silica aerogel is difficult but feasible, even if a layer of aerogel remains around the dust particle, and we have set up a methodology based on Raman and SERS analysis for adenine mapping on individual dusts of adenine-doped serpentinite. Two different adenine signatures were obtained, possibly due to the presence of the “free” adenine that redissolved in solution, and physisorbed adenine on serpentinite. These promising results show that further studies are essential for the development of a database of SERS spectra of prebiotic molecules for space mission return samples, or for the development of onboard SERS for future space missions. As a matter of fact, the miniaturization and automation of Raman instruments and the optimization of the robustness of SERS analysis (SERS substrate, etc.) could enable the development of onboard devices for space missions [30,31].

## 4. Materials and Methods

### 4.1. Serpentinite

To reproduce the Tanpopo capture experiment, model extraterrestrial dusts were designed. The serpentine mineral was chosen to mimic the inorganic matrix. The serpentinite used comes from Mont Chenaillet [32] and was identified by X-ray diffraction and Raman spectroscopy as the polymorph lizardite (Table S2, Figure S5) [33,34,35]. Lizardite has the composition Mg_3_Si_2_O_5_[OH]_4_ and contains about 13 wt% H_2_O. 

Serpentinite rock was first broken with a hammer on an aluminum plate into small pieces, which were then placed in a planetary mill. After grinding, the powders were sieved, and the portions recovered and used had the following diameters: between 50 and 100 µm, and between 100 and 150 µm.

### 4.2. Adenine Solutions

Adenine (≥99%) and MgCl_2_ (for molecular biology, 1.00 ± 0.01 mol/L) were purchased from Sigma-Aldrich (St. Louis, MO, USA). A 5·10^−3^ mol/L stock solution of adenine was prepared in water, heated at 90 °C (1 h to ensure dissolution) and diluted as necessary. For the most concentrated solutions, the adenine concentration was checked with UV-visible spectroscopy. 

### 4.3. Adenine Doping of Serpentinite

To reproduce the Tanpopo capture experiments in space, model extraterrestrial dusts have been designed. Adenine-doped serpentinite particles were prepared through two different processes. In the first process, 20 mg of serpentinite powder was incubated with 480 µL of aqueous solutions of adenine. After 2 h, the pellet was centrifuged and placed on a glass slide and left to dry under air at room temperature. Maximum amount of Adenine adsorbed on serpentinite are presented in Table S1. The effective amount of adenine adsorbed was estimated by TGA (Thermogravimetric Analysis) on the sample prepared with the highest amount of adenine (5·10^−3^ mol/L) and was estimated to be 9.3% (*w*/*w*). Since the serpentinite fraction used has a specific surface area of 8 m^2^/g, we can estimate a surface density of about 0.9 adenine molecules per nm^2^, which is not far from the physical monolayer. These samples are generally used for preliminary batch analyses. 

Doped serpentinite samples (10^−4^ mol/L in adenine) were heated until 300 °C to monitor the effect of temperature on organic doping. A few mg was deposited in a quartz or alumine cup and heated in a furnace oven Pyrox (up to 1000 °C/air) for 2 h prior to analysis.

A second, slightly different procedure is used for doping the dust that will be used for LGG projection. Approximately 20 mg of serpentine powder was added to approximately 5 mL of a 5·10^−3^ mol/L or 5·10^−6^ mol/L adenine solution and stirred at room temperature for 12 h. The adenine-doped particles were collected by centrifugation. The particles were washed twice with about 5 mL of pure water, collected by centrifugation, and then dried in a freeze-dryer.

Here, we use the term of “doping”, which is common in astrochemical experiments. In other contexts, these procedures could be referred to as adenine deposition—a term that does not prejudge the type or strength of interactions established between adenine and the mineral surface.

### 4.4. Aerogel Preparation

The hydrophobic aerogel blocks with the Tanpopo flight model quality were manufactured as described by Tabata et al. [36]. Aerogel blocks were prepared in a contamination-controlled environment in a facility in Chiba University [37].

### 4.5. Inclusion of the Dust in the Aerogel with Light Gas Gun Projection

Hypervelocity impact experiments using the two-stage LGG at ISAS/JAXA were conducted to simulate the intact capture of micrometeoroids using the same type of aerogel onboard the Tanpopo–ISS experiment.

The adenine-doped serpentine dust of 50–150 µm in average size were filled into a 7.1 mm-diameter polycarbonate cylinder called a sabot. The sabot was accelerated to about 6.2 km/s by the LGG under a vacuum of ~10 Pa. The sabots were separated after releasing into a free flight injection tube, and the dust filled inside the sabot were accelerated and flown toward the Tanpopo aerogel target. The aerogel which captured the ejected microparticles had a 0.01 g/cm^3^ density and was approximately 15 × 15 × 20 mm in size; it was placed at the end of the vacuum chamber so that the center of the injection tube was aligned by the center of the aerogel block. The decompression and recompression operations of the LGG vacuum chamber were performed slowly so as not to deform the aerogel. The penetration depth of the impacted mineral dust into the aerogel was approximately 3–5 mm. After the recovery of the aerogel block from the LGG chamber, the aerogel around the particles was cut out with a sharp blade (Figure 2). The samples were placed on aluminum foil and stored in the folded foil.

### 4.6. Creighton’s Colloid Synthesis

All the chemicals used were purchased from Sigma-Aldrich (St. Louis, MO, USA, purity ≥ 99%). Silver colloids were obtained by the gradual addition of a 100 mL silver nitrate solution (10^−3^ mol/L) to a 300 mL sodium borohydride solution (2·10^−3^ mol/L) under strong agitation at a low temperature (in a cool ice bath) in the dark [25]. The yellow solution obtained exhibits an absorption band at around 400 nm (measured on a Cary 3 UV-visible spectrometer, Varian, Victoria, Australia) and a Zeta potential of −35 mV (Malvern ZetaSizer, Malvern, UK).

### 4.7. Raman Instrumentation

Raman spectroscopy measurements were conducted using a LabRam HR800 (Horiba-Jobin Yvon, Longjumeau, France) instrument characterized as having a focal length of 800 mm, a 600 lines/mm grating and the Rayleigh line was filtered by using Ultra Law Frequency Bragg filters. The analyses were performed with the 514 nm excitation wavelength of a water-cooled Ar^+^ laser. The detector consists of a CCD camera with a Peltier effect cooling system. The spectral resolution was about 4 cm^−1^ and the calibration was checked with respect to the 520.5 cm^−1^ silicon band. The laser power was adjusted according to the sample (from 50 µW to 2 mW at the sample) and the counting time was from 5 to 30 s.

### 4.8. Mapping

Spectra were acquired with the LabSpec6 software (Horiba Jobin Yvon, Longjumeau, France), which allows an automated mapping acquisition by a motorized XY microscope stage. Raman maps were acquired by using a 100× LWD (long working distance) objective. For each sample, multiple (more than two) Raman or SERS maps were recorded to verify the reproducibility and sample stability with time. 

### 4.9. SERS Analysis

#### 4.9.1. Doped Serpentinite Clusters

In the first step, the analyses were carried out on sets of several dozen doped serpentinite dusts which were referred to as “clusters” in the following sections. These analyses on clusters allow optimizing the measurement conditions before carrying out the analysis on an individual particle. 

A few mg of doped serpentinite was taken with a spatula and deposited on a gold mirror. Then, 3 to 5 μL of premixed silver colloid with salt (45 μL silver colloid, 5 μL MgCl_2_, final concentration of MgCl_2_ 10^−2^ mol/L) was added. Focus was made on the dust/silver colloid interface (using the breathing mode of adenine around 730 cm^−1^, and the O-H stretching mode of water molecules around 3425 cm^−1^). Spectra were registered as a function of time (acquisition time around 5 s, 3 times) as the SERS effect evolves with time and especially with water evaporation. The most significant spectra are presented in Figure 1. The laser power was adjusted depending on the analyzed sample to prevent heating and burning, unless it is intended to carbonize the sample for reference purposes. 

#### 4.9.2. Individual Doped Serpentinite Particles

The samples were analyzed prior to projection in the aerogel. The dust particles were deposited on a gold mirror and dusts were separated with a single paint brush hair under a binocular. In addition, 0.5 μL of silver colloid was then added (premix: 45 μL of silver colloid, with 5 μL of MgCl_2_, final concentration of MgCl_2_ 10^−2^ mol/L). SERS spectra were recorded after 15 min of incubation.

#### 4.9.3. Individual Doped Serpentinite Particles after Aerogel Extraction

The analysis sequence is detailed in Figure 2C. The samples were prepared using the following steps: the addition of 0.5 μL of methanol/water solution (MeOH/H_2_O; 80/20) to chemically modify the aerogel and expulse the dust (incubation for about 10 min), prior to the addition of 0.5 μL of silver colloid (premix: 45 μL of silver colloid, with 5 μL of MgCl_2_, final concentration of MgCl_2_10^−2^ mol/L). Raman mapping was performed before colloid addition and after 15 min of incubation. As a reference, dust without adenine was also analyzed, after the projection and extraction steps.

## Data Availability

All data and materials are available on request from the corresponding author. The data are not publicly available due to ongoing researches using a part of the data.

## Supplementary Materials

The following supporting information can be downloaded at: https://www.mdpi.com/article/10.3390/gels10040249/s1, Figure S1: Representative SERS spectra obtained on individual doped serpentinite dust (5·10^−3^ mol/L and 5·10^−6^ mol/L, diameter between 50 and 100 µm) (after colloid addition) prior projection in the aerogel, Figure S2: Raman mapping of a doped serpentinite dust with 5·10^−6^ mol/L of adenine after projection/extraction of the aerogel before and after MeOH/H_2_O treatment (before colloid addition), Figure S3: Superposition of all the Raman spectra obtained by the mapping shown in Figure 4, corresponding to the doped serpentinite dust (5·10^−6^ mol/L) (after MeOH/H_2_O treatment and colloid addition), Figure S4: ATR-FTIR of adenine powder as a function of heating temperature of the sample. Adenine powder was exposed to each temperature for two hours, Figure S5: X-ray diffraction pattern of the serpentinite sample (A); Raman spectrum of serpentinite (B), Table S1: Maximum amount of adenine adsorbed on serpentinite, with the specific surface area measured for the serpentinite used of 8 m^2^/g, Table S2: Main bands observed in Raman Lizardite spectra with their assignments.
