# Supplementary material for: Surface-Enhanced Raman Spectroscopy (SERS) for Identifying Traces of Adenine in Organic-Bearing Extraterrestrial Dust Analogs Captured in the Tanpopo Aerogel after Hypervelocity Impacts
Source: Gels. 2024 Apr 6;10(4):249. doi: 10.3390/gels10040249 (PMC11049422; doi:10.3390/gels10040249)
Supplement: Supplementary file 1 [file gels-10-00249-s001.zip › gels-2883809-supplementary.pdf]

gels

Article

Surface-Enhanced Raman Spectroscopy (SERS) for Identifying Traces of Adenine in Organic-Bearing Extraterrestrial Dust Analogs Captured in the Tanpopo Aerogel after Hypervelocity Impacts

Aline Percot [1,*], Farah Mahieddine [1], Hajime Yano [2], Sunao Hasegawa [2], Makoto Tabata [3], Akihiko Yamagishi [4], Hajime Mita [5], Alejandro Paredes-Arriaga [6,7], Marie-Christine Maurel [8], Jean-François Lambert [9], Donia Baklouti [10] and Emilie-Laure Zins [1]

Supplementary Materials

Figure S1: Representative SERS spectra obtained on individual doped serpentinite dust (5.10^{-3} mol/L and 5.10^{-6} mol/L, diameter between 50 and 100 µm) (after colloid addition) prior projection in the aerogel. P.2

Figure S2: Raman mapping of a doped serpentinite dust with 5.10^{-6} mol/L of adenine after projection/extraction of the aerogel before and after MeOH/H_2O treatment (before colloid addition). P.3

Figure S3: Superposition of all the Raman spectra obtained by the mapping shown in Figure 4, corresponding to the doped serpentinite dust (5.10^{-6} mol/L) (after MeOH/H_2O treatment and colloid addition). P.4

Figure S4: ATR-FTIR of adenine powder as a function of heating temperature of the sample. Adenine powder was exposed to each temperature for two hours.

Figure S5: X-ray diffraction pattern of the serpentinite sample (A); Raman spectrum of serpentinite (B). P.6

Table S1: Maximum amount of adenine adsorbed on serpentinite, with the specific surface area measured for the serpentinite used of 8 m^2/g. P.7

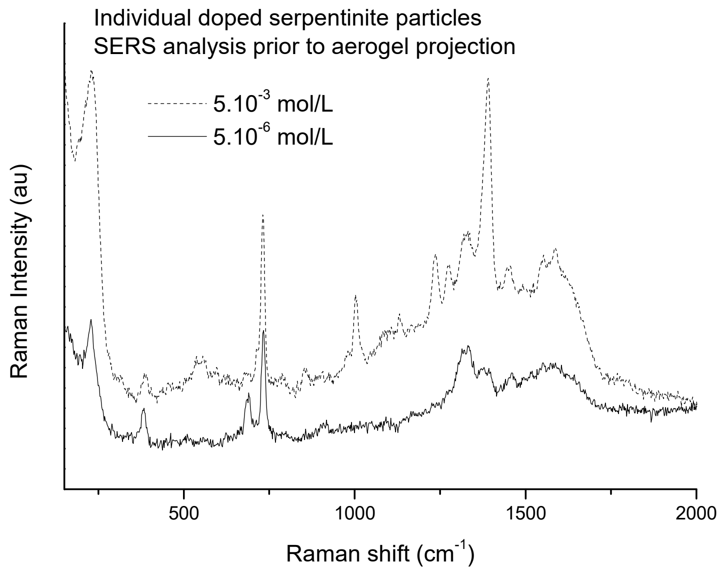

Figure S1: Representative SERS spectra obtained on individual doped serpentinite dust (5.10^{-3} mol/L and 5.10^{-6} mol/L, diameter between 50 and 100 µm) (after colloid addition) prior projection in the aerogel.

gels

MDPI

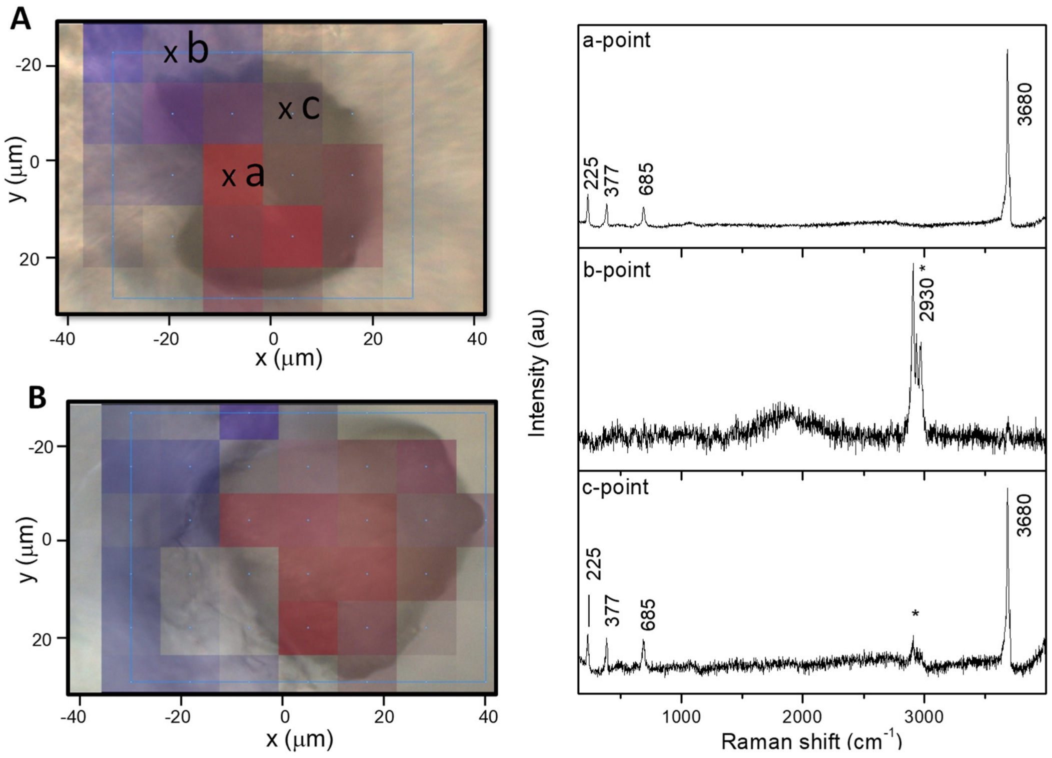

Figure S2: Raman mapping of a doped serpentinite dust with 5.10^{-6} mol/L of adenine after projection/extraction of the aerogel before and after MeOH/H_2O treatment (before colloid addition). (A) Optical microscope image of dust superimposed with two distributions of compounds extracted from Raman mapping; the red map corresponds to intensity integration around the 3680 cm^{-1} band (associated to serpentinite); the blue map corresponds to intensity integration around the 2930 cm^{-1} bands (associated to aerogel). a) Characteristic spectrum related to serpentinite (a-point indicates the pixel from which this spectrum is derived). b) Characteristic spectrum related to the aerogel (b-point indicates the pixel from which this spectrum is derived). The overlay of the red map with the dust confirms its chemical nature (i.e. serpentinite), while the blue map shows the area covered by aerogel before expulsion, i.e. present over the entire surface of the sample analyzed. The spectrum c obtained at point c in image A is the sum of spectra a and b (i.e. the simultaneous presence of serpentinite and aerogel*). (B) Optical microscope image of the same dust after MeOH/H_2O treatment. Using a new Raman map, the same compound distribution extraction as in A is superimposed (i.e. serpentinite (red) and aerogel* (blue)). These distributions show the expulsion of the dust from the aerogel (it is no longer present over the entire area analyzed).

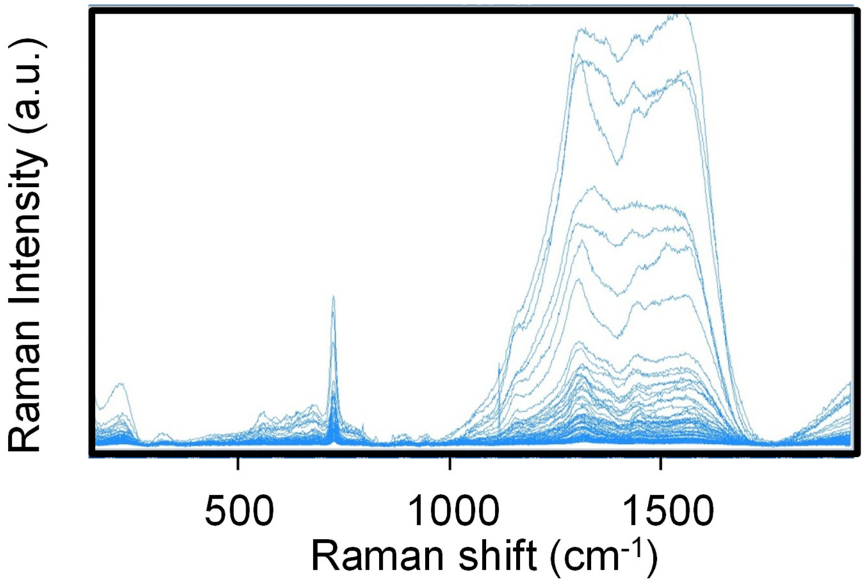

Figure S3: Superposition of all the Raman spectra obtained by the mapping shown in Figure 4, corresponding to the doped serpentinite dust (5.10^{-6} mol/L) (after MeOH/H_2O treatment and colloid addition). A baseline treatment was applied. Raman spectra show the adenine markers at 730 and 1330 cm^{-1}.

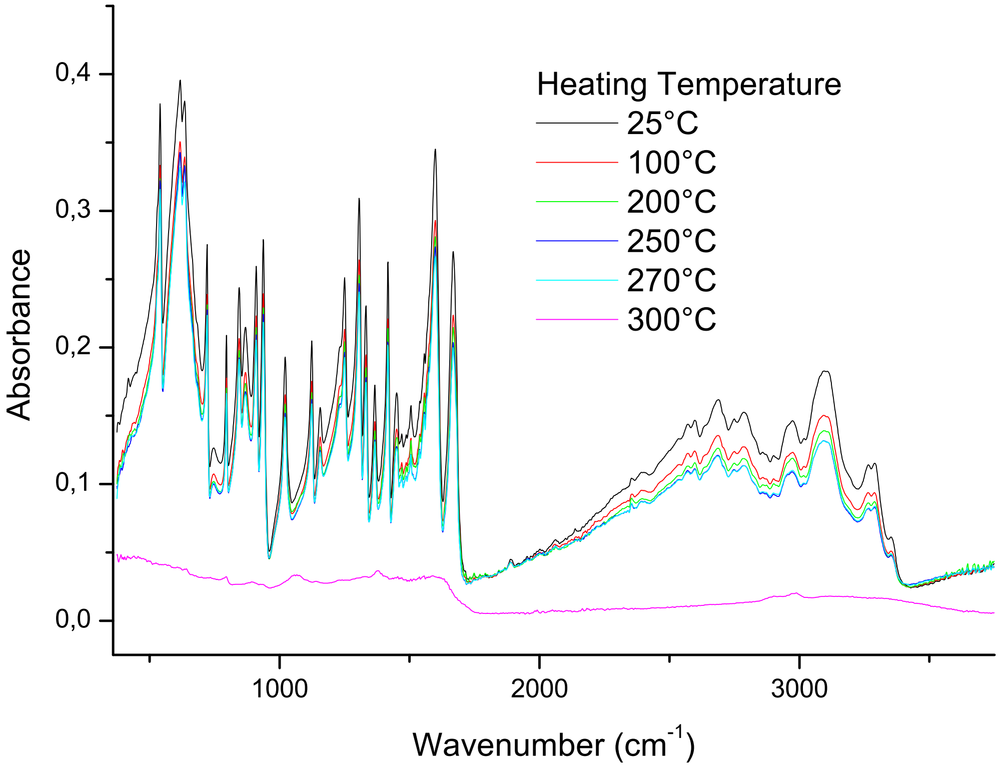

Figure S4: ATR-FTIR of adenine powder as a function of heating temperature of the sample. Adenine powder was exposed to each temperature for two hours.

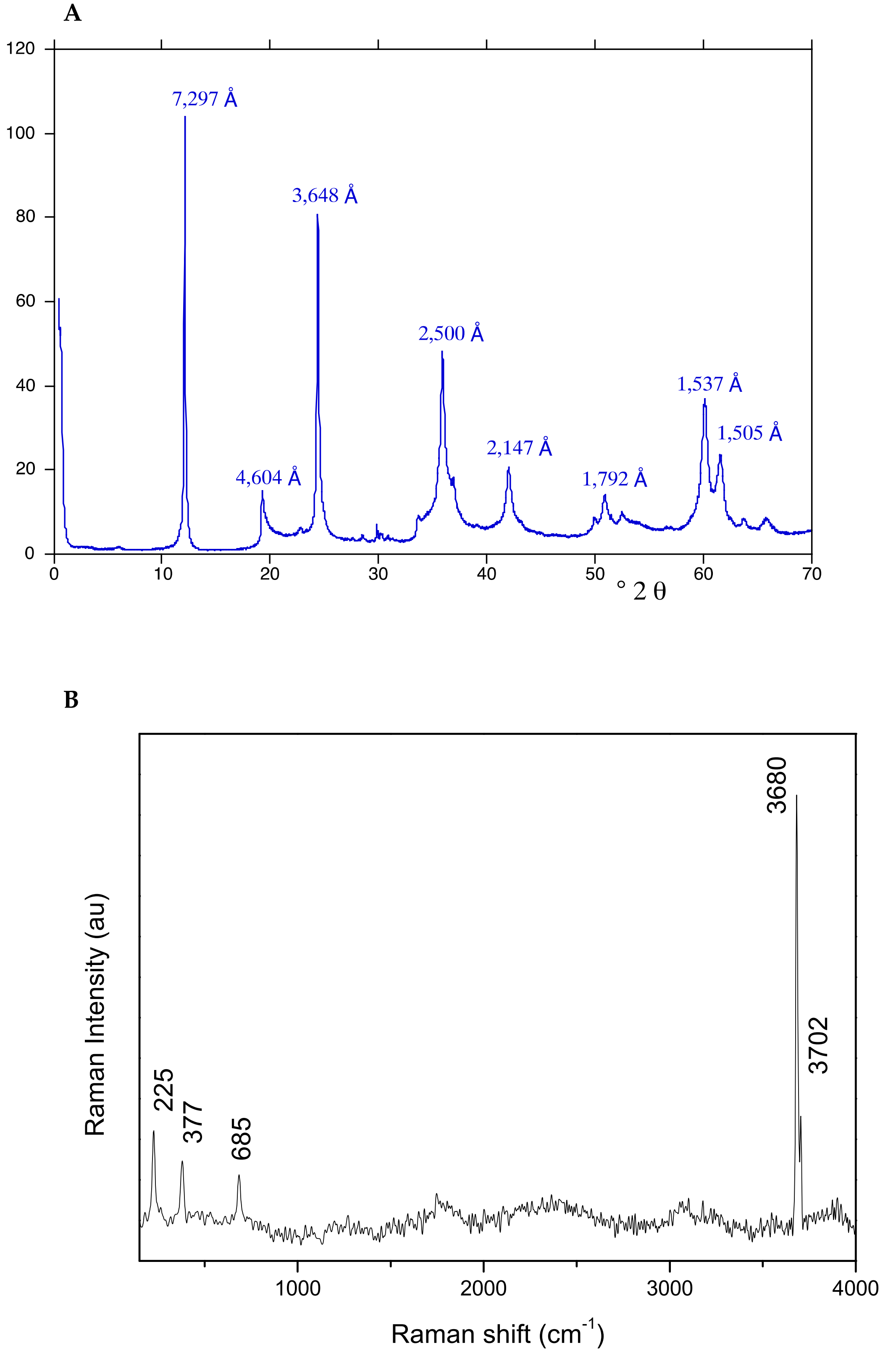

Figure S5: X-ray diffraction pattern of the serpentinite sample (A); Raman spectrum of serpentinite (B).

gels

MDPI

Adenine concentration in deposition solution (mol/L)	Maximum adenine loading (if 100% adsorbed) (mg/g serpentinite)	Maximum adenine loading (molecule/nm^2)
5.10^{-3}	1.62	9
5.10^{-4}	$1.62.10^{-1}$	9.10^{-1}
5.10^{-5}	$1.62.10^{-2}$	9.10^{-2}
5.10^{-6}	$1.62.10^{-3}$	$9;10^{-3}$
5.10^{-7}	$1.62.10^{-4}$	9.10^{-4}
5.10^{-8}	$1.62.10^{-5}$	9.10^{-5}
5.10^{-9}	$1.62.10^{-6}$	9.10^{-6}

Table S1: Maximum amount of adenine adsorbed on serpentinite, with the specific surface area measured for the serpentinite used of 8 m^2/g.

Raman (cm^{-1})	Assignments [1–3]
~225 s	unassigned vibration of O-H-O groups (involving OH in ditrigonal cavity)
~380 s	Sym ν_5 of SiO_4
~690 s	ν_S of Si-O_b-Si
~3660, 3680 vs, 3700 vs	ν_{OH} in "outer" OH of octahedral sheet

Table S2: Main bands observed in Raman Lizardite spectra with their assignments.

1. Auzende, A.-L.; Daniel, I.; Reynard, B.; Lemaire, C.; Guyot, F. High-Pressure Behaviour of Serpentine Minerals: A Raman Spectroscopic Study. *Phys. Chem. Miner.* **2004**, *31*, 269–277. https://doi.org/10.1007/s00269-004-0384-0.
2. Rinaudo, C.; Gastaldi, D.; Belluso, E. Characterization of Chrysotile, Antigorite and Lizardite by Ft-Raman Spectroscopy. *Can. Mineral.* **2003**, *41*, 883–890. https://doi.org/10.2113/gscanmin.41.4.883.
3. Petriglieri, J.R.; Salvioli-Mariani, E.; Mantovani, L.; Tribaudino, M.; Lottici, P.P.; Laporte-Magoni, C.; Bersani, D. Micro-Raman Mapping of the Polymorphs of Serpentine. *J. Raman Spectrosc.* **2015**, *46*, 953–958. https://doi.org/10.1002/jrs.4695.
